# 

**Published:** 1899-03

**Authors:** 


					﻿LEADS VETERINARY JOURNALISM IN AMERICA.
Vol. XX.
MARCH, 1899.
No. &
PUBLISHED MONTHLY AT S3 A YEAR. SINGLE COPIES, 30 CENTS.
FOREIGN SUBSCRIPTIONS J3.50 PER YEAR.
THE JOURNAL
OF
Comparative Medicine
AND
VETERINARY ARCHIVES
EDITED AND PUBLISHED BY
RUSH SHIPPEN HUIDEKOPER, Veterinarian (Alfort),
W. HORACE HOSKINS, D.V.S.,
H. D. GILL, V.S.
ALL COMMUNICATIONS AND PAYMENTS SHOULD BE ADDRESSED TO
Dr. W. Horace Hoskins, Managing Editor, 3452 Ludlow St., Philadelphia, Pa.
Copies may be had on order of all news-dealers at home and abroad.
AN ADVERTISING MEDIUM UNEQUALLED IN THE VETERINARY FIELD.
				

